# Predictors of mental health among U.S. adults during COVID-19 early pandemic, mid- pandemic, and post-vaccine eras

**DOI:** 10.1186/s12889-024-17781-x

**Published:** 2024-02-29

**Authors:** Niloofar Ramezani, Bruce G. Taylor, Elizabeth Flanagan Balawajder, Kai MacLean, Harold A. Pollack, John A. Schneider, Faye S. Taxman

**Affiliations:** 1 Department of Biostatistics, Virginia Commonwealth University, Box 980032, One Capital Square, 830 East Main St, Richmond, VA 23219 USA; 2 NORC at the University of Chicago, Public Health Department, 4350 East West Highway, 8th floor, Bethesda, MD 20814 USA; 3 Crown Family School of Social Work, Policy, and Practice, Department of Public Health Sciences, Urban Health Lab, University of Chicago, 969 E 60th St, Chicago, IL 60637 USA; 4 Department of Medicine and Public Health Sciences, Crown Family School of Social Work, Policy, and Practice, University of Chicago, 5841 South Maryland Avenue MC 5065, Chicago, IL 60637 USA; 5 Schar School of Policy and Government, George Mason University, 3351 Fairfax Drive Van Metre Hall, Arlington, VA 22201 USA

**Keywords:** COVID-19, Mental health, Race, Precautionary measures

## Abstract

**Background:**

A collective trauma like COVID-19 impacts individuals differently due to socio-contextual and individual characteristics. Younger adults, minorities, affiliates of certain political parties, and residents of some regions of the United States reported experiencing poorer mental health during the pandemic. Being diagnosed with COVID-19, or losing a friend/family to it, was related to more adverse mental health symptoms. While the negative impact of COVID-19 on health outcomes has been studied, mental health changes during this pandemic need further exploration.

**Methods:**

In a study of 8,612 U.S. households, using three surveys collected from a nationally representative panel between May 2020 and October 2021, using a repeated cross-sectional design, a linear mixed effect regression model was performed to investigate factors associated with the mental health status, based on the Mental Health Inventory-5, of individuals throughout different phases of the COVID-19 pandemic, and whether an improvement over time, especially after vaccines became available, was observed.

**Results:**

An overall improvement in mental health was observed after vaccines became available. Individuals with no COVID-related death in their household, those not wearing masks, those identifying as members of the Republican Party, race/ethnicities other than Asian, men, older adults, and residents of the South were less likely than others to report mental health challenges.

**Conclusions:**

Our results highlight the need for widespread mental health interventions and health promotion to address challenges during the COVID-19 pandemic and beyond. Due to the worse mental health observed among Asians, younger adults, women, low-income families, those with a higher level of concern for COVID-19, people who lost someone to COVID-19, and/or individuals with histories of opioid use disorder and criminal legal involvement, over the period of this study, targeted attention needs to be given to the mental health of these groups.

## Background

COVID-19 has influenced almost every facet of life since March 2020, when the World Health Organization (WHO) declared a global pandemic. Besides physical and economic wellbeing, the impact on mental health has been a key concern since the earliest days of the pandemic [1]. Data from March and April 2020 demonstrated increased psychological distress including anxiety, depression, insomnia, and suicidal ideation compared to before the pandemic [2]. Specifically, a study using the Patient Health Questionnaire-9 (PHQ-9) among adults in the United States and United Kingdom found that psychological distress was three to four times higher in April 2020 compared to similar studies before the COVID-19 pandemic in 2018 and 2019 [2]. Similarly, a study of U.S. adults found that depression was approximately three times higher in April 2020 than before the pandemic while generalized anxiety was 10–15 times higher [3].

Some studies note that mental health status declined several months into the pandemic after having initially increased rates. For example, Daly and Robinson found that psychological distress increased between March 10 and April 14, 2020, but by June 2020, it had decreased to mid-March levels [4]. Another study of 150,000 Americans found that while anxiety initially increased and then returned to baseline prevalence after the first few months of the pandemic, sadness and depression levels continued to increase through July 2020 [5]. Mental health challenges persisted later in the pandemic, as the percentage of adults reporting anxiety or depression increased from 36% in August 2020 to 41.5% in February 2021 [6].

The pandemic has impacted the mental health of people differently based on a variety of factors. A review of literature from early in the pandemic suggests “layers” of potential stress can contribute to one’s mental health status [2]. While everyone is affected by the outermost layer, or the stress of living through the pandemic, fewer people are affected by an inner layer of caring for someone with COVID, or the innermost layer, which is contracting severe COVID-19. The more layers of stress that a person experiences, the more deleterious their mental health outcomes may become [2]. Higher levels of vicarious stress were observed during this pandemic among health care workers and general public due to their exposure to others’ physical and psychological sufferings [7]. Significantly higher vicarious traumatization scores were observed in the general public and medical staff, especially non-front-line nurses, under the situation of the spread and control of COVID-19. Therefore, Li et al. urged the implementation of the early strategies that aim to prevent and treat vicarious traumatization in medical staff and general public during this pandemic [7]. This current study, not only looks into the early stages of the COVD-19 pandemic, but it also looks into the post-vaccine Omicron era and investigates any changes in the mental health of the U.S. population through October 2021.

### COVID-19 and mask wearing

When COVID-19 pandemic started, public health agencies prominently recommended that Americans wear face masks to prevent COVID-19 transmission, with estimates of mask wearing among the population varying throughout the life of the pandemic. In a poll from October 2020, 93% of adults reported wearing a mask often or always [8]. In July 2021, only half reported wearing a mask “every time” or “most of the time” in indoor settings [9]. In a nationally representative survey conducted from April 30-May 4, 2020, the peak COVID-19 incidence within the U.S., 73% of respondents reported mask wearing, with women more likely to report protective behaviors than men, as were those over age 60. Respondents who self-identified as having low incomes, histories of criminal justice involvement, and Republican Party affiliation, were less likely to report four protective behaviors, including mask wearing [10]. Perceptions and behaviors related to the pandemic have shown to have associations with mental health, with those who take more protective behaviors, such as wearing a mask, reporting poorer mental health [11, 12].

One study identified groups based on risk and protective behavior taking, grouping individuals into “minimally” (25.5%), “moderately” (35.5%), and “highly” (28.8%) protective groups as well as a “risky” (12.1%) group [12]. The minimally protective group typically endorsed wearing a mask, but no other behaviors, while the highly protective group highly endorsed all protective behaviors including wearing a mask, washing hands often, taking supplements for immunity, and isolating from people outside their household, among others [12]. The study found that anxiety and post-traumatic stress disorder (PTSD) were higher among the “risky” group and the “moderately” and “highly” protective groups, meaning that it was lowest among the “minimally” protective group [12]. This group did not endorse all protective behaviors, but also did not engage in risky behaviors. Thus, high anxiety or PTSD may lead to more risky behavior and greater adherence to COVID-19 prevention behaviors, like masking [12]. Interestingly, in a comparison of mental health in China and the United States, mask wearing was associated with poorer mental health in the U.S. but had the opposite effect in China [13].

### Opioid use and criminal legal histories

The opioid epidemic continued to ravage the U.S. during the entire period of COVID-19 pandemic with over 77,000 opioid-related overdose deaths recorded in calendar year 2021 alone [14]. Concern was raised that those with an opioid use disorder (OUD) were more likely to experience mental health decline during COVID-19 due to their unique experiences and challenges. For example, those who use opioids may face greater stressors and subsequent mental health difficulties due to COVID-related risk factors. Those who use opioids are more likely to develop COVID-19 due to multiple comorbidities, including a history of high rates of psychological trauma and other mental health conditions, and are more likely to be affected by general COVID-related stressors like a loss of income, isolation, housing instability, and general anxiety [13, 15, 16]. Individuals who use opioids may also experience greater anxiety and depression due to COVID-19 related restrictions, such as lack of access to drug supplies or clean substance use supplies, disruption of drug markets, the inability to obtain needed medication and treatment, and the closing of substance use treatment centers, which can not only affect individual mental health but increase the risk for overdose and/or death [16–18]. People who use opioids can experience the dual stigma from both using opioids and contracting COVID-19, which may exacerbate their feelings of exclusion, prevent them from seeking care for either condition, and ultimately worsen their mental health [19]. Most respondents who supported increasing COVID-related resources for people experiencing homelessness or people with low income did not suggest such resources for those who used drugs [20]. Such complications cause mental health distress for this population. They may also result in more lethal overdoses due to social isolation. For example, visits to emergency departments were significantly higher March– October 2020 for opioid overdoses compared to the same period in 2019 [21].

People who have a history of criminal legal involvement (CLI) are another group that is likely to experience mental health vulnerabilities. Individuals who were recently arrested or were under probation or parole were more likely to seek treatment for mental health concerns compared to those with no such history [22]. Having a CLI history was significantly associated with experiencing a major depressive episode in the past year [23]. To date, there are no known studies directly examining how the mental health of people with a history of opioid use or CLI has been affected through the various stages of the COVID-19 pandemic. Therefore, we explored the novel risk factors for poor mental related to prior criminal legal involvement (prior history of conviction for a misdemeanor, or felony crime, or being incarcerated in jail or prison) and misuse of opioid/prescription pain medications in a nationally representative sample.

### Sociodemographic factors

Mental health status differs based on sociodemographic factors, including age, income, and race. Specifically, mental health improves with age. During the COVID-19 period, the prevalence of anxiety and depression declined with increasing age, with age appearing to moderate the effects of COVID-19 on anxiety [24, 25]. Those with a lower income experienced poorer mental health during the pandemic, including more clinical symptoms of anxiety and depression [26]. All individuals reported decreased wellbeing at the onset of the pandemic, but those living with a romantic partner had better wellbeing on average, suggesting that relationships served as a protective factor [27], with the specific characteristics of relationship appearing to play an important moderating role [28].

On average, Black, Hispanic, and Asian American populations appear to have experienced poorer mental health compared to White individuals throughout the pandemic into 2021. Data from the 2020 Healthy Minds Study indicate that over a quarter of students who identified as Asian American or Pacific Islander (AAPI) experienced racial/ethnic discrimination in the context of COVID-19 and over two-thirds of AAPI students reported a mental health condition [29]. Racial discrimination was associated with greater odds of having depression, anxiety, binge drinking, self-injury, and suicidal ideation [29].

One study of Black and Asian American individuals in May-July 2020, in the early stages of the COVID-19 pandemic, found that experiences of vicarious racism, or witnessing racism against one’s racial group, were associated with more depression and anxiety [30]. Similarly, it was suggested in a study from April and May 2020 that 13% of adults identifying as multiracial and races other than Hispanic, Black, or White worried about experiencing discrimination and blame for spreading COVID-19 [31]. Those identifying as Hispanic have also been found to have higher rates of depression and substance use than White populations during COVID-19 [30]. In this study, we look into how the mental health of people of different race/ethnicity, sex, age groups, and income level changed over the three timepoints of the study.

### Political party

Given the polarized nature of U.S. politics, political party affiliation has been shown to be associated with practice and uptake of preventive behaviors against COVID-19 [32]. One study of students in the South found that being affiliated with the Republican Party was significantly and negatively associated with vaccine acceptance [33], and that right-leaning political affiliation and having a higher proclivity towards risk were predictors of vaccine hesitancy [34]. People who lean more Republican practice fewer preventive behaviors compared to Democrats or Independents [32]. A lower percentage of Republicans who were at risk of severe COVID-19 wore a mask, and were less likely to consider themselves at risk of COVID-19 [35]. Independents or Republicans were significantly less likely to be fearful of COVID-19 [36]. While several studies have emerged to demonstrate links between political identification and COVID-related behaviors, few have specifically assessed whether one’s identification with a specific political party was related to mental health outcomes over the period of this pandemic. Balawajder et al. investigated this issue but with only one wave of data, rather than over multiple timepoints during this pandemic [11].

### The current study

Our study augments the literature by examining self-reported mental health of the U.S. population at three specific times during the COVID-19 pandemic: early pandemic (May 2020), the pre-vaccine heart of the pandemic (October 2020), and the post-vaccine Omicron era (October 2021). In addition to examining mental health across a representative population, we identify specific vulnerable subgroups whose mental health was reported to be more impacted during the pandemic.

## Methods

### Sample

As part of a repeated cross-sectional study, the same survey was administered to different cohorts of U.S. adults over three consecutive timepoints. The first survey was administered in May 2020 (with *n* = 1,002 adults). The next survey took place in October 2020 (with *n* = 1,095 adults), and the third survey was administered in October 2021 (with *n* = 6,515 adults). A repeated cross-sectional design was used to ensure a higher sample size [37]. This design does not require the same individuals being followed over time, yet, due to using the same population at all measurement times, allows us to study the population changes over time [38, 39]. The use of different samples at each timepoint is common in the study of large populations to ensure a higher sample size without having to follow the same cohort over time as is done in longitudinal studies [37]. Appropriate methodology needs to be employed to produce unbiased estimates while using this design [40].

Participants were drawn from a random sample of the AmeriSpeak® panel. AmeriSpeak® is a probability-based ongoing panel of about 40,000 households designed to be representative of the U.S. household population (excluding those not found in households such as individuals currently incarcerated, institutionalized, and homeless). The AmeriSpeak participants are selected through a stratified random sample of U.S. households using area probability and address-based sampling, with a known, nonzero probability of selection from the National Opinion Research Center (NORC) national sample frame. Sampled households are contacted by regular mail, telephone, and field interviewers (face-to-face) to capture harder-to-reach cases. About 97% of the U.S. household population is statistically represented through AmeriSpeak’s sampling approach [41]. Details about AmeriSpeak methods can be found in https://amerispeak.norc.org/us/en/amerispeak/about-amerispeak.html. With an annual panel retention rate of about 85% [41], methodology research on AmeriSpeak shows only minor differences, on average under 1.5%, by sex, age group, race/ethnicity, education, marital status, employment, income, region, and home Internet access compared to the U.S. Census American Community Survey [42, 43]. AmeriSpeak’s weighted household recruitment rate, which includes a second stage of recruitment for initial non-responders to capture harder-to-reach populations is 37%, one of the highest for comparable national probability-based household panels [44].

For this paper, randomly-selected panel members were invited to participate in this study via email, with reminders sent via email and phone. For each wave, about 25–40% of the contacted participants from the AmeriSpeak panel of invited adult panelists completed this project’s survey. Considering our first stage of sampling (with a 37% panel recruitment rate), the completion rate at the second sampling stage for each of the three waves was 25%, 27%, and 40%, respectively. The October 2021 survey was a larger survey with a somewhat better response rate due to some extra weeks in the field to collect the data from the larger group. The same approach was used in all three surveys regarding emailing and calling the respondents on the phone to remind them to do the survey.

To account for different sample sizes and representativeness at each wave of survey administration, data are weighted to national census benchmarks, taking into account selection probabilities (balanced by sex, age, education, race/ethnicity, and region) and nonresponse [41]. We used weighted data for our analyses, and took the complex design of the survey into account. Making use of a response propensity approach in calculating the conditional probability that a particular respondent completed the survey given observed covariates, we used the standard validated approach to nonresponse weights developed by Bethlehem et al. in creating the weights used in this study [45]. Weighting adjusted for selection probabilities and nonresponse, thereby rendering results representative of the household population. These weights helped addressing participation biases from known characteristics.

### Variables

Mental Health Inventory 5 (MHI-5) was used as a screener to measure mood, depression and anxiety disorders [46]. With items on psychological wellbeing, the MHI-5 was developed for the general population [47] and has high levels of internal consistency (0.80 to 0.96) [48]. Details about this response variable and the following predictor variables can be found in Table 1.

Predictors used in our model were COVID-19 mask wearing, COVID-related death in the household, social stigma toward people with OUD, history of opioid misuse, history of CLI, and demographics. These demographic and background variables were respondent’s age, sex, race/ethnicity, income, education, marital status, residential region, and their political party. Predictors were measured and assessed in all three time points. First, they were used to predict the mental status of the individuals at each timepoint and then to model average changes in the mental health status of people over time.

**Table 1:** Variables used in this study

Variables used in this study	Variable and answer categories	Variable details
MHI-5 (Response Variable)	The scale includes 5 items asking the respondent how much of the time in the last month he/she had considered himself/herself to be: 1. a very nervous person 2. had felt downhearted 3. had felt calm and peaceful 4. had felt so down in the dumps that nothing could cheer them up 5. considered themselves to be a happy person. The answers were scored on six-point scales ranging from “All of the time” to “None of the time.” Two items were reverse coded before computing the scale mean ranging from 5 to 30. The higher the score the better the respondents’ mental health.	Mental Health Inventory 5 (MHI-5) was used as the response variable to measure mood, depression and anxiety disorders [46]. With items on psychological wellbeing, the MHI-5 was developed for use with the general population [47]. The internal consistency of the original tool ranged from 0.80 to 0.96, as reported in several studies in the general population [48].
COVID-19 Mask Wearing	People were asked whether they wore a mask when leaving home in response to the outbreak of coronavirus. Answer Categories: (1) Yes (2) No	To assess concern for COVID-19, participants were asked to indicate whether they engaged in protective behaviors and risky behaviors.
COVID-Related Death in the Household	Respondents were asked whether anyone in their household passed away or died of causes related to COVID-19 related causes. Answer Categories: (1) Yes (2) No	This question was asked to assess respondents’ personal experience with COVID-related trauma.
Social Stigma Toward People with an Opioid Use Disorder Scale	Questions asked about 1. willingness to have a person with a past history of OUD work with you or marry into your family 2. willingness to have a person with a current OUD work with you or marry into your family 3. perceived dangerousness and trustworthiness of people with OUD. Respondents rated their agreement with each statement on a five-point Likert-type scale (1 = strongly disagree, 2 = somewhat disagree, 3 = neither disagree nor agree, 4 = somewhat agree, and 5 = strongly agree). Four items on this scale were reverse coded before computing the mean of all six items.	We developed a 6-item social stigma scale (Cronbach’s alpha = 0.78) adapted from prior stigma survey research [49, 50]. A higher score on our stigma scale reflects greater stigma toward individuals with an OUD.
History of Opioid Misuse	We asked respondents “Have you ever used opioids/prescription pain medication illicitly obtained or used in a way not prescribed by a doctor?” Answer Categories: (1) Yes (2) No	This question was asked to measure the respondent’s personal experience with opioid misuse. Opioid misuse was defined for the respondent as use of opioids or prescription pain medication illicitly obtained or used in a way not prescribed by a doctor.
History of Criminal Legal Involvement	Respondents were asked whether they ever had a conviction for a misdemeanor, or felony crime, or been incarcerated in jail or prison. Answer Categories: (1) Yes (within a year), (2) Yes (over a year), (3) No	This question was asked to measure the respondent’s personal experience with the criminal legal system.
Age	Answers categorized into 7 categories of 18–24, 25–34, 35–44, 45–54, 55–64, 65–74, and 75 + years old	
Sex	Answers: Male and Female	
Race/Ethnicity	Categorized into 6 categories of White, Black, Hispanic, Asian, 2 + non-Hispanic, and other	
Income	Answers included 4 categories of Less than $30,000, $30,000 to under $60,000, $60,000 to under $100,000, $100,000 or more	
Education	Answers included 4 groups of <high school (HS), HS/GED, Some College/Associate Degree, and College Graduate	
Marital Status	Answers included 6 groups of Married, Widowed, Divorced, Separated, Never married, and Living with a partner	
Census Region the Respondent Lived in	Answers included 4 groups of Northeast, Midwest, South, and West	
Political Party That the Respondent Most Strongly Identified with	Answers included 6 categories of Democrat, Lean Democrat, Don’t Lean/Independent/None, Lean Republican, Republican, and Unknown	

### Analysis

Descriptive statistics and multiple time-point Analysis of Variance (ANOVA), followed by post-hoc tests, were used to assess differences in self-reported mental health status over time. Different sample sizes at the three waves are adjusted for in all analyses, so the results are not biased by the larger sample size in Wave 3 versus Waves 1 and 2. To identify factors associated with the overall changes in mental health status of the U.S. population over time, we performed a linear mixed effect regression model, which is an extension of linear regression, adding random effect to account for the non-independence of our data [51]. Considering the different cohorts sampled at each wave of the study, we could not measure the individual growth/change; instead, we measured the overall change in mental health status of our population over time using the linear mixed effect model. Mental health (MHI-5), measured at three times, was used as the response/outcome variable, and predictors were selected for inclusion based on *a priori* hypothesis and previous findings. Statistical analyses were conducted in R [52] with a significance level of $\alpha =0.05$. Multiple-test correction was used to minimize type I error.

## Results

**Fig. 1:**
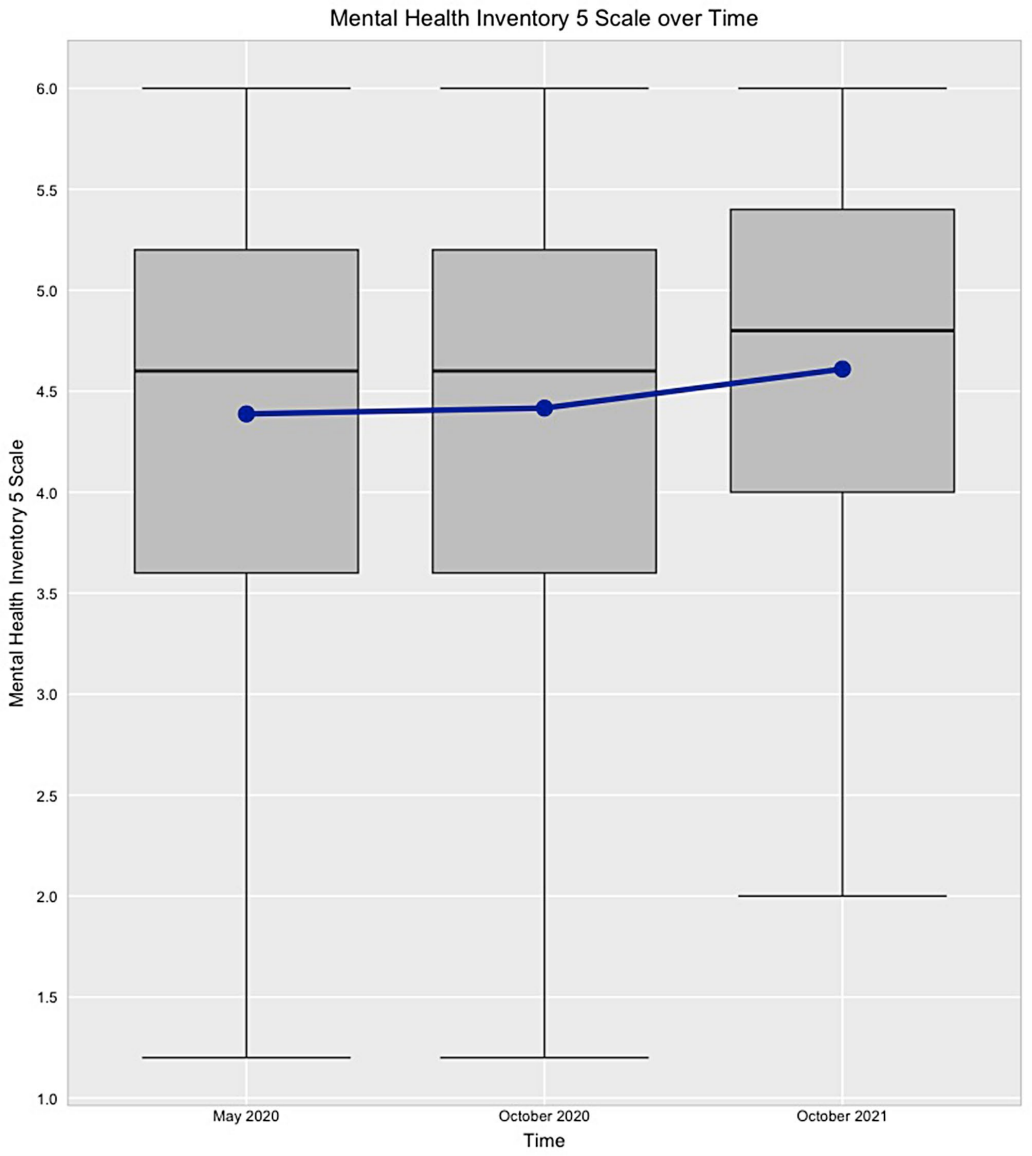
Boxplots showing Mental Health Inventory (MHI-5) change of the U.S. households over time during COVID-19 pandemic (means are shown as connected dots)

Average mental health scores (MHI-5) improved significantly over time from May 2020 through October 2021. The mean (and standard deviation) for the mental health scale in May 2020, October 2020, and October 2021 are 4.39 (1.05), 4.42 (1.06), and 4.61 (1.01), respectively. The overall increase of the mental health score of the U.S. households over time is shown in Fig. 1, suggesting improved mental health over time.

First, a multiple time-point ANOVA found significant change of mean mental health status over time (F(2, 8560) = 32.52, *P* <.001). Second, to understand where the pairwise differences lie across the three waves, Tukey’s honestly significant difference (HSD) post-hoc test was performed. Table 2 shows that the significant increase in the mean MHI-5 score happened post-vaccine era in October 2021 (*P* <.001 for May 2020 versus October 2021; *P* <.001 for October 2020 versus October 2021). Significant variables, at the significance level of 0.05, are bolded in Tables 2 and 3.

**Table 2:** Pair-wise post-hoc mean comparison of mental health (MHI-5) over time

	(I) time	(J) time	Mean Difference (I-J)	Std. Error	Sig.	95% Confidence Interval	
						Lower Bound	Upper Bound
Post-hoc	**May 2020**	Oct. 2020	− 0.02902	0.04494	0.812	− 0.1390	0.0810
		**Oct. 2021**	− 0.22234	0.03482	**< 0.001**	− 0.3076	− 0.1371
	**Oct. 2020**	May 2020	0.02902	0.04494	0.812	− 0.0810	0.1390
		**Oct. 2021**	− 0.19332	0.03358	**< 0.001**	− 0.2755	− 0.1111
	**Oct. 2021**	**May 2020**	0.22234	0.03482	**< 0.001**	0.1371	0.3076
		**Oct. 2020**	0.19332	0.03358	**< 0.001**	0.1111	0.2755

Finally, to understand what factors predict the change of mental health status over the period of this study, a linear mixed effect model was performed. Within this model, we considered the sampling weights and adjusted for different sample sizes of the three waves to ensure results are not affected by the higher sample size in wave 3. This model scales the data across different waves to ensure different sample sizes are accounted for and therefore the results and estimates across different timepoints are comparable [53]. Weights, which were explained earlier, adjusted for selection probabilities and nonresponse. The mixed effect model allowed us to account for the correlation that exists among measurements taken at each timepoint as if time is a cluster of the individuals whose measures are taken at that timepoint, while looking into overall changes over time. It also accounts for unbalanced data. This method allowed us to identify significant factors that were associated with the overall mental health status of the U.S. population over time. As shown in Table 3, time is significant showing that over time, mental health improved with people doing the worst at the beginning of pandemic (May 2020); this is consistent with the ANOVA results (Table 2). People in the third timepoint of the study reported doing significantly better than the early stage of COVID (*p* <.001).

People who did not wear masks reported better mental health compared to people who wore masks over time (*p* <.001). Overall, the mean mental health score (MHI-5 score) is 4.56 for mask wearers and 4.61 for individuals not wearing a mask during the entire period of this study. To be more specific, within timepoint 1, mean MHI-5 is 4.36 for mask wearers, which is lower than 4.47 which is the mean MHI-5 score for people who did not wear a mask. Within timepoint 2, these mean scores are 4.41 versus 4.49 for people wearing masks compared to the individuals not wearing a mask, respectively. The trend stays the same within timepoint 3, with mask wearers having an average MHI-5 score of 4.60 compared to the mean of 4.78 which belonged to the group who did not wear a mask. Individuals who did not have a COVID-related death in their household reported better mental health than people who did (*p* <.001). The difference in the mental health score was higher between these two groups with the average MHI-5 score of 3.92 for individuals who had a COVID-related death in their household compared to the mean MHI-5 score of 4.57 for people who did not experience this. To be more specific, the mean MHI-5 of people who had a COVID-related death in their household (versus the other group) was 3.17 (versus 4.4), 3.42 (versus 4.43), and 4.18 (versus 4.61) within timepoints 1, 2, and 3, respectively.

Regarding vulnerable populations, people not using opioids reported better mental health compared to opioid users (*p* <.001). Overall, people who had used opioid reported a lower average MHI-5 score of 4.35 compared to people who did not use opioid, who had an average MHI-5 score of 4.61 throughout the entire period of this study. Considering that we did not have MIH-5 measures for the individuals who used opioid prior to the COVID-19 pandemic, we cannot be sure if they started at a worse mental health state compared to the rest of the U.S. population. We observed that they had a lower mental health score (mean = 3.83) compared to the others (mean = 4.43) at the early stage of the pandemic (timepoint 1). Therefore, if opioid users had a lower mental health score prior to the pandemic, the difference between the two groups may not have been only due to the COVID-19 pandemic. However, at timepoint 3, and after the availability of vaccines, which is when the mental health of the overall population starts improving, the difference between the two groups became smaller with the opioid users having a mean MHI-5 score of 4.23 compared to the non-opioid users who had the mean MHI-5 score of 4.65. People who had been incarcerated reported worse mental health, with people who have been incarcerated within the past year having a lower mental health score compared to individuals who were incarcerated over a year ago or individuals who were never incarcerated (*p* =.007 and 0.002, respectively).

Race, age, sex, marital status, political party, and residential region were all significant predictors of mental health score over time. Asians reported the worst mental health status during the pandemic with Black and His panic respondents reporting significantly better scores (*p* <.001) and p = .048, respectively). The younger the respondents were, the worse they were doing in terms of mental health (18–24 age category was doing worse than 25–30 age category, 25–30 age category was doing worse than 35–44 age category, and so on, with 75 + year-olds reporting the best scores). Men reported better mental health than women (*P* <.001), and all marital status categories reported better mental health status than individuals living with an unmarried partner.

Respondents identifying as Republican reported better mental health than Democrats or Independents (*P* <.001). People living in the South of the U.S., even though not statistically significant, reported better mental health over time (during the COVID-19 era) compared to other regions, with Northeasterners reporting the worst mental health. Households with less than $30,000 income reported the worst mental health state (*P* <.001).

**Table 3:** Mixed-effects model predicting mental health (MHI-5) over time

Parameter	Estimate	Std. Error	Sig.	95% Confidence Interval	
				Lower Bound	Upper Bound
Intercept	4.334	0.130	< 0.001	4.078	4.590
**Time/Wave 1** (Compared to Time/Wave 3) ^a^	− 0.185	0.035	**< 0.001**	− 0.253	− 0.117
Time/Wave 2 (Compared to Time/Wave 3) ^a^	0.044	0.044	0.317	− 0.042	0.130
**Not wearing mask** (Compared to wearing mask)	0.144	0.038	**< 0.001**	0.069	0.219
**Opioid Use: Yes = 1** (Compared to No = 0)	− 0.233	0.036	**< 0.001**	− 0.304	− 0.163
**Incarceration: Incarcerated within a year (past year)** (Compared to no Incarceration)	− 0.335	0.125	**0.007**	− 0.581	− 0.089
**Incarceration: Incarcerated over a year ago** (Compared to no Incarceration)	− 0.113	0.037	**0.002**	− 0.187	− 0.040
**No household COVID death** (Compared to having household COVID death)	0.649	0.098	**< 0.001**	0.456	0.842
Stigma scale score	0.004	0.003	0.156	− 0.002	0.010
Race/Ethnicity = White (Compared to Asian)	0.032	0.051	0.524	− 0.067	0.132
**Race/Ethnicity = Black** (Compared to Asian)	0.268	0.059	**< 0.001**	0.152	0.384
Race/Ethnicity = Other (Compared to Asian)	0.108	0.111	0.330	− 0.109	0.326
**Race/Ethnicity = Hispanic** (Compared to Asian)	0.108	0.055	**0.048**	0.001	0.215
Race/Ethnicity = 2 + non-Hispanic (Compared to Asian)	0.095	0.081	0.241	− 0.064	0.255
**Income = Less than $30,000** (Compared to >$100,000)	− 0.187	0.035	**< 0.001**	− 0.255	− 0.119
Income=$30,000 to under $60,000 (Compared to >$100,000)	− 0.010	0.032	0.751	− 0.073	0.053
**Income=$60,000 to under $100,000** (Compared to >$100,000)	0.094	0.031	**0.002**	0.033	0.155
Education = No HS diploma (Compared to BA or above)	− 0.026	0.043	0.544	− 0.110	0.058
Education = HS graduate or equivalent (Compared to BA or above)	− 0.010	0.030	0.730	− 0.069	0.048
Education = Some college (Compared to BA or above)	− 0.043	0.028	0.121	− 0.098	0.011
**Age = 18–24** (Compared to Age = 75+)	− 0.864	0.060	**< 0.001**	− 0.982	− 0.745
**Age = 25–34** (Compared to Age = 75+)	− 0.817	0.053	**< 0.001**	− 0.916	− 0.709
**Age = 35–44** (Compared to Age = 75+)	− 0.723	0.051	**< 0.001**	− 0.824	− 0.622
**Age = 45–54** (Compared to Age = 75+)	− 0.564	0.051	**< 0.001**	− 0.665	− 0.464
**Age = 55–64** (Compared to **Age** = 75+)	− 0.288	0.049	**< 0.001**	− 0.385	− 0.191
**Age = 65–74** (Compared to Age = 75+)	− 0.131	0.050	**0.009**	− 0.228	− 0.033
**Sex = Male** (Compared to Females)	0.212	0.022	**< 0.001**	0.170	0.255
**Marital Status = Married** (Compared to Living with partner)	0.227	0.046	**< 0.001**	0.138	0.317
**Marital Status = Widowed** (Compared to Living with partner)	0.182	0.072	**0.012**	0.041	0.323
**Marital Status = Divorced** (Compared to Living with partner)	0.142	0.053	**0.008**	0.038	0.247
**Marital Status = Separated** (Compared to Living with partner)	0.197	0.068	**0.004**	0.064	0.330
Marital Status = Never married (Compared to Living with partner)	0.025	0.046	0.585	− 0.065	0.116
**Region = Northeast** (Compared to West)	− 0.095	0.034	**0.005**	− 0.161	− 0.029
Region = Midwest (Compared to West)	− 0.001	0.033	0.980	− 0.065	0.064
Region = South (Compared to West)	0.018	0.029	0.530	− 0.038	0.074
Political Party = Unknown (Compared to Republican)	0.039	0.167	0.816	− 0.288	0.366
**Political Party = Democrat** (Compared to Republican)	− 0.336	0.029	**< 0.001**	− 0.394	− 0.278
**Political Party = Lean Democrat** (Compared to Republican)	− 0.342	0.038	**< 0.001**	− 0.416	− 0.267
**Political Party = Don’t Lean, Independent, none** (Compared to Republican)	− 0.178	0.033	**< 0.001**	− 0.243	− 0.112
**Political Party = Lean Republican** (Compared to Republican)	− 0.209	0.040	**< 0.001**	− 0.287	− 0.131

^a^ Time/Wave 1 = May 2020, Time/Wave 2 = October 2020, and Time/Wave 3 = October 2021

## Discussion

COVID-19 has had a disparate impact on various populations in the United States. This study illustrates how these patterns changed over three periods of COVID-19: early in the pandemic, mid- pandemic (or the pre-vaccine heart of the pandemic era), and post-vaccine Omicron periods. Asian-Americans, Democrats and lean-Democrats, vulnerable populations such as individuals with OUD or who have recent CLI, and residents of the Northeast reported worse mental health status than other subgroups. These patterns are relatively stable in terms of the mental health status of individuals during the three aforementioned periods of the pandemic, and over time. More importantly, this study illustrates how vulnerable populations are more likely to report lower mental health functionality than the general population during the pandemic. Interestingly, individuals that lean or identify as Republican tend to report better mental health over this period, irrespective of the phase of the pandemic. This could be due to less concern for risk among this group, meaning that since they are not as concerned about risk as the other groups, they feel less anxiety, depression, etc. The data indicate that people who were less affected by COVID-19 or paid less attention to the masking protocols reported better mental health status.

Low-income households reported more adverse mental health symptoms. Their poorer mental health status could be due to how their and their family members’ health was impacted by the COVID crisis and/or due to the higher impact that the COVID-19 pandemic likely extends into their lives through secondary effects on employment levels, poverty, social inequality, and more [54]. Therefore, attention to low-income households could help the wellbeing of these members of the community.

The COVID-19 pandemic created havoc on people routines and lifestyle and created a variety of economic and social hardships. The Conservation of Resources Theory [55] offers a framework to understand responses to these stressors from COVID-19 and suggests that stress results from circumstances involving threatened or actual loss of valued resources. These resources reflect what one values in terms of objects, states, and conditions; the loss of these types of resources will drive individuals into higher levels of stress and anxiety [55]. It is the desire to defend, conserve, and acquire valued resources which drives human behavior in the face of these stressors [55]. This theory helped us understand how large proportions of the population had to adapt to a new changed reality from COVID-19 (e.g., lockdowns) and the more changes they experienced in their daily routines, for example in the earlier days of COVID-19, the stronger was their anxieties in terms of COVID-19.

The patterns found in this study illustrate the importance of a person’s wellbeing in terms of mental health during emergency situations such as pandemics. Those that were in a better state at the beginning of the pandemic, tended to remain so. Those that were inflicted with various social problems such as CLI or OUD tended to continue to have problems, and were more likely than most groups to report adverse mental health consequences of the COVID pandemic. But it also appears that those that reside in the Northeast and who identify as Democrats also tend to be more impacted by COVID in terms of lower reported mental health scores.

The question remains as to how to continue to improve mental health through the pandemic, especially for vulnerable populations. That is, those who are adopting prevention practices such as mask wearing should be able to do so without suffering setbacks in their mental health. Prevention education around this issue could help mask wearers to better address their mental health, and to perhaps find some relief of their mental health symptoms knowing that they are taking the precautionary measures.

Individuals who are more anxious and concerned about COVID-19 might take more preventive steps to protect themselves and others from accompanying illness. Unfortunately, due to the lack of pre-pandemic data on our survey items, anxiety measures were not recorded pre and post pandemic; therefore, we could not directly evaluate the changes in mental health of individuals with anxiety pre- and post-pandemic, or whether individuals with higher levels of anxiety (pre-pandemic) remained at the same level of anxiety post-pandemic and as a result of their anxiety, they opted for taking more preventive steps. For the same reason, we could not measure how mental health status for different sociodemographic factors, including age, income, and race categories compared post-pandemic to the pre-pandemic era, so our results and conclusions focus on mental health of U.S. households during COVID-19.

Although this study did not explore issues related to available services to help individuals address their mental health needs, the crisis in terms of the mental health workforce and availability of programs and services, and how many of these services limited operations and switched to telehealth or on-line services, may impact our findings. Future studies should explore the impact of service availability on the mental health of individuals facing additional service barriers, like people with OUD. Our study highlights the need for more resources to address mental health needs of U.S. households. Our study adds to the literature by showing how mental health declined in the U.S. population when the COVID-19 pandemic started throughout 2020, but mental health started improving in 2021. We think that this improvement could be partially due to improved resilience in mental health in response to the pandemic, and possibly the renewal of hope for the return to normalcy because of the emergence and availability of vaccines to general public. Future studies can look into this hypothesis.

### Limitations

First, our data are based on self-reported mental health status rather than through direct clinical examination. Second, our response rates are modest. We weighted our data using the national census benchmarks, considering selection probabilities and nonresponse to better match the population. This helps with the generalizability of our results to the entire population of the U.S [41]. Third, because different cohorts are sampled at each wave of the study, we could not measure individual-level growth/change in mental health; instead, we measured the overall change in mental health status of our population over time using the linear mixed effect model. Finally, since we did not have pre-pandemic data on our survey items, anxiety measures were not recorded pre and post pandemic; therefore, we could not directly evaluate the changes in mental health of individuals with anxiety pre- and post-pandemic.

## Conclusion

The COVID-19 pandemic posed generational physical and mental health challenges globally. Considering the substantial increase in distress in the U.S. during the COVID-19 crisis, mental health must be assessed in each pandemic phase and over time, as secondary pandemic effects extend to employment, poverty, social inequality, and more [54].

Our findings explore the mental health of a representative national sample of adults in the United States from early pandemic, through mid-pandemic, and into post-vaccine era (Wave 3), which is when mental health started improving. This improvement could be partially due to improved resilience in mental health in response to the pandemic, and/or the renewal of hope for the return to normalcy because of vaccines.

Sustained attention to mental health remains necessary to address wide-ranging pain caused by mental illness [56] and to guide policy during the pandemic and beyond [2, 57]. Our findings emphasize the serious mental health implications of the pandemic, particularly for those who experienced or witnessed the most serious impacts of COVID-19. Our results highlight the need for widespread mental health interventions and health promotion to address these challenges during the COVID-19 pandemic and beyond with targeted attention to the mental health needs of Asians, younger adults, women, low-income families, those who lost a loved one to COVID-19 or have a higher level of concern for this disease, or individuals with histories of OUD and CLI.

## Data Availability

De-identified data used and/or analyzed during the current study are available on reasonable request. Data requests should be sent to Dr. Bruce Taylor at taylor-bruce@norc.org.

## Abbreviations

OUD: Opioid use disorder
CLI: Criminal legal involvement
MHI-5: Mental Health Inventory 5
ANOVA: Analysis of Variance
HSD: Tukey’s honestly significant difference
